# Phenolic Compounds from Apples: From Natural Fruits to the Beneficial Effects in the Digestive System

**DOI:** 10.3390/molecules29030568

**Published:** 2024-01-23

**Authors:** Lidija Jakobek, Petra Matić

**Affiliations:** Faculty of Food Technology Osijek, Josip Juraj Strossmayer University of Osijek, Franje Kuhača 18, HR 31000 Osijek, Croatia; petra.matic@ptfos.hr

**Keywords:** stomach, small intestine, colon, phase II metabolites, catabolites

## Abstract

Conditions in the gastrointestinal tract and microbial metabolism lead to biotransformation of parent, native phenolic compounds from apples into different chemical forms. The aim of this work was to review current knowledge about the forms of phenolic compounds from apples in the gastrointestinal tract and to connect it to their potential beneficial effects, including the mitigation of health problems of the digestive tract. Phenolic compounds from apples are found in the gastrointestinal tract in a variety of forms: native (flavan-3-ols, phenolic acids, flavonols, dihydrochalcones, and anthocyanins), degradation products, various metabolites, and catabolites. Native forms can show beneficial effects in the stomach and small intestine and during the beginning phase of digestion in the colon. Different products of degradation and phase II metabolites can be found in the small intestine and colon, while catabolites might be important for bioactivities in the colon. Most studies connect beneficial effects for different described health problems to the whole apple or to the amount of all phenolic compounds from apples. This expresses the influence of all native polyphenols from apples on beneficial effects. However, further studies of the peculiar compounds resulting from native phenols and their effects on the various parts of the digestive tract could provide a better understanding of the specific derivatives with bioactivity in humans.

## 1. Introduction

Phenolic compounds are intensively studied for their bioactivities in the human organism [1,2,3]. These studies show their strong potential for various beneficial effects. However, the forms in which they are present in certain parts of the organism are important for their activity.

In the digestive tract, for example, phenolic compounds can be biotransformed due to environmental conditions that include different pH values or the activity of different enzymes, which cause changes in the chemical structures of the phenolic compounds. These biotransformations are important for the beneficial effects of these compounds since the actual forms of phenolic compounds present are the ones that have the potential for bioactivities. Importantly, the digestive tract is the first system that comes in contact with phenolic compounds from food. Bioactivities in the digestive tract can be the basis of their beneficial effects in many diseases. That is why the fate of phenolic compounds in the digestive tract, including their amounts, forms, and bioactivities, should be better known.

It is well known that in the digestive tract, phenolic compounds are found in their native form, as they are in food [4,5,6]. These are parent compounds that are subjected to various different conditions in different phases of digestion (pH) and can change into different degradation products. Naturally present microbiota in the colon can additionally transform phenolic compounds into various catabolites [7]. Phenolic compounds or their catabolites undergo further phase I metabolism (hydrolysis, oxidation, and reduction) and phase II metabolism (glucuronidation, sulfation, and methylation), the result of which are many forms of metabolites [8,9]. These metabolites pass into the liver where additional biotransformation occurs with further such reactions. However, enterohepatic recirculation can send some of the metabolites back into the intestinal lumen [8,9,10]. All these mentioned forms (native phenolic compounds, products of degradation, catabolites, and metabolites) can be found in the digestive tract depending on the phase of digestion. And they have the potential to be active compounds. Their potential bioactive effects are still not completely known.

Due to the common consumption of apples in a normal diet, apples can be an important source of phenolic compounds [11,12]. The identification of forms of phenolic compounds from apples present in the digestive tract can show which particular forms have the potential to exhibit bioactivities. Additional studies should enable better connection of beneficial effects to specific forms of phenolic compounds or phenol subgroups present in the digestive system.

The aim of this paper is to review the current knowledge of the phenolic compounds from apples, their forms present in different parts of the digestive tract (mouth, stomach, small intestine, and colon) and their potential beneficial effects.

## 2. Apples

Apples belong to family Rosaceae, genus *Malus*, and species *Malus domestica*. Their origins are in Asia. Apples are consumed worldwide during the whole year which makes them an important source of nutrients. Water takes up the highest portion in apples, with approximately 80% of the whole mass [11]. Other compounds in apples included in their nutritional value are proteins and carbohydrates. As an example, various traditional and exotic apples contain, within 100 g of fresh weight, 0.07–0.08 g of proteins, around 11 g of sugars, and around 3 g of fibers [13]. Similarly, commercial varieties of apples contain 0.2–0.3 g/100 g fw of proteins, 12.9–13.3 g/100 g fw of total sugars, and 2.4–2.6 g/100 g fw of total dietary fibers [11].

Besides proteins and carbohydrates, apples may contain various vitamins such as vitamin C, vitamins from the B complex, or vitamin E [13]. Traditional and exotic apples contain vitamin C (with averages of 8.88 and 8.28 mg/100 g fw for traditional and exotic varieties, respectively) and vitamin E (with averages of 176 and 141 μg/100 g fw for traditional and exotic varieties, respectively) [13]. Many minerals such as potassium are also common. In old cultivars of apples, the main macroelement was indeed potassium (in peel 104–158 and in flesh 74–93 mg/100 g fw) [12]. Likewise, in the previously mentioned study, potassium was a mineral found in the highest amount (average 101 and 99 mg/100 g fw in traditional and exotic varieties, respectively) [13]. In addition to potassium, apples contain other macroelements such as P, Mg, and Ca [12]. The main microelements in apples are Fe, Al, B, and Na [12,13]. Common organic acids are citric and L-malic acids [13]. The amounts of organic acids and sugars are important because their appropriate ratio gives a pleasant taste to apples, which is essential for consumer acceptance.

A class of compounds from apples that are intensively studied because of their bioactive effects are phenolic compounds. Even though apples are not the fruit with the highest amounts of phenolic compounds, they are an important source of phenolic compounds, due to their availability throughout the whole year and their common consumption in the diet.

## 3. Phenolic Compounds from Apples

**Figure 1:**
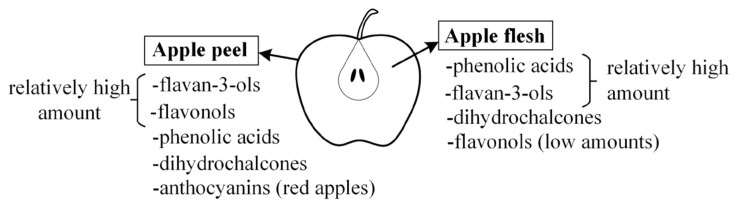
Main subgroups of phenolic compounds from apples.

Apples contain five main subgroups of phenolic compounds (Figure 1)—flavan-3-ols (oligomeric procyanidins, procyanidin B1, procyanidin B2, procyanidin B3, procyanidin B4, procyanidin C1, (+)-catechin, and (−)-epicatechin), dihydrochalcones (phloretin-2′-glucoside, and phloretin-2′-xyloglucoside), phenolic acids (chlorogenic acid, neochlorogenic acid, *p*-coumaroylquinic acid, cryptochlorogenic acid, and protocatechuic acid), flavonols (galactoside, glucoside, rutinoside, xyloside, arabinoside, rhamnoside of quercetin, and kaempferol glycosides), and anthocyanins (cyanidin-3-glucoside, cyanidin-3-galactoside, and others) [12,13,14,15,16,17,18,19,20,21].

Phenolic compounds have different distributions of amounts in the peel and in the flesh. In addition to flavan-3-ols and phenolic acids, the peel of red apples usually contains anthocyanins and high amounts of flavonols [12,15]. On the other hand, the flesh usually does not contain anthocyanins, or their content is low [15], except for some varieties that are characterized as having red flesh [22]. Likewise, the amount of flavonols is low in the flesh. However, the flesh has high amounts of phenolic acids [15] and relatively high amounts of flavan-3-ols and dihydrochalcones.

This distribution of phenolic compounds in the flesh or peel can be significant for the beneficial effects of apples. Due to various treatments of apples during their growth, commercial varieties of apples are often eaten without the peel. Accordingly, classes of phenolic compounds from apple flesh such as flavan-3-ols, phenolic acids, or dihydrochalcones can become more important for the potential positive effect in the digestive tract. On the other hand, for apples grown organically, which can be consumed with the peel, phenolic compounds from the peel can be important. In that case, apart from flavan-3-ols, phenolic acids, and dihydrochalcones, flavonols can show the potential for beneficial effects.

## 4. Phenolic Compounds from Apples in the Digestive Tract

The release and presence of phenolic compounds from apples in the digestive tract is the basis of their beneficial effects. Many in vitro and in vivo studies suggest the presence of relatively high amounts of phenolic compounds from apples in all parts of the digestive tract [5,6,23,24,25,26]; however, these amounts are still lower than the natural amounts in apples (Table S1, Supplementary Materials).

More specifically, according to in vitro simulated digestion [4,5,6], phenolic compounds are released in the mouth, stomach, and small intestine in lower amounts than the native amounts in apples. In the stomach, total phenolic compounds are released at from 29 to 47 mg/100 g fw (fresh weight), and in the small intestine from 18 to 27 mg/100 g fw, while the native amounts are higher, namely, 36–52 mg/100 g fw in four different apple varieties [5]. Likewise, for in vitro digestion, in a study of the amounts of three phenolic compounds—procyanidins, quercetin-3-rutinoside, and phloretin-2′-glucoside—from the flesh and peel of five apple varieties, before digestion, the amounts of these compounds in apples were found to be up to 79, 44, and 68 mg/100 g fw, respectively [6]. After the digestion process, the released amounts of the same phenolic compounds were as follows: in the mouth, up to 28, 15, and 18; in the stomach, up to 32, 29, and 27; and in the small intestine, up to 44, 21, and 31 mg/100 g fw, respectively [6]. These values are lower than the native amounts.

The study of human subjects also shows that relatively high amounts of phenolic compounds from apples can be found in the digestive tract, but those amounts are still lower than the natural amounts in the fruit [26]. In a study where ileal fluid from ileostomy subjects was analyzed, for subjects who consumed apple juice, 42% of ingested total phenolic compounds was recovered in the ileal fluid. This represents the amount that could be found in the small intestine and could eventually reach the large intestine [26] where these compounds can be degraded by the activity of microbiota into various catabolites. Furthermore, the percentage of catabolites found after colonic fermentation of apples is 29 to 47% of the initial amount in apples [27].

According to the mentioned studies, even though the amounts of released phenolic compounds in the mouth, stomach, small intestine, and colon are lower than the amounts in apples, the amounts are still significant (Table S1) and important for bioactive effects.

### 4.1. The Forms of Phenolic Compounds in the Digestive Tract

The forms of phenolic compounds from apples that are present in the digestive tract vary according to the different parts of the digestive tract. These differences arise from environmental conditions such as pH. The pH in the stomach is acidic, around 1–2.5 [28,29]. It increases in the proximal small intestine (pH 6.6) and reaches pH 7.5 in the terminal ileum. pH shows a sharp fall to 6.4 in the cecum and increases from the left to the right colon (mean value 7.0) [28]. Similar values of pH or a similar trend were reported in Fallingborg [29] and Nugent et al. [30]. At different pH values, phenolic compounds can be stable or in some cases degrade.

#### 4.1.1. Mouth and Stomach

**Figure 2:**
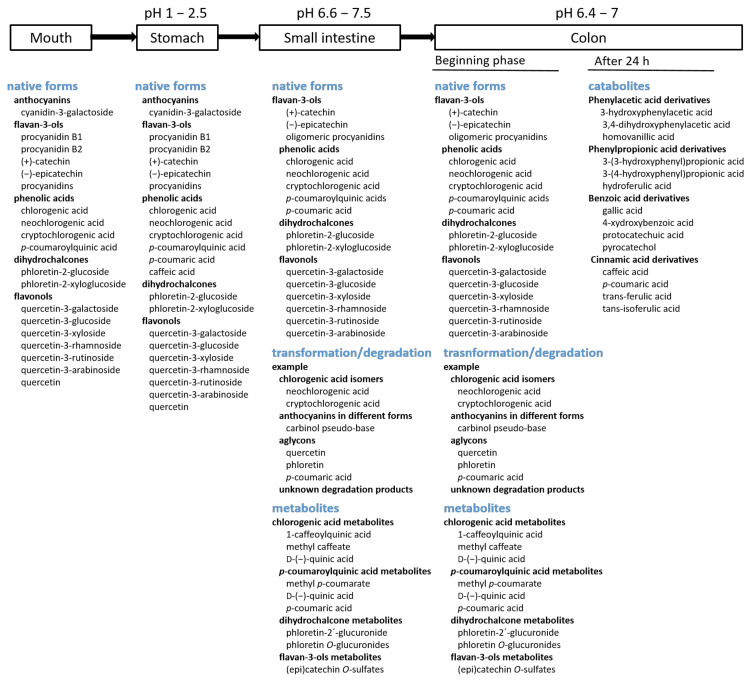
Forms of phenolic compounds from apples in the digestive tract and some examples of individual compounds reported in earlier studies [5,6,11,23,24,26,31,32,33].

In the mouth and stomach (Figure 2), phenolic compounds from apples are present in their native form.

According to the in vitro digestion of apples [5,23,24], flavan-3-ols, phenolic acids, dihydrochalcones, flavonols, and anthocyanins are released in the mouth and stomach from the apple matrix where they stay in their native, unchanged form. Similarly, in the study of Tenore et al. [6], after the in vitro simulated digestion of apple peel and flesh where only procyanidins, quercetin-3-rutinoside, and phloretin-2′-glucoside were studied, phenolic compounds were stable in the gastric phase [6].

Food stays in the mouth for only a short time period. However, it stays in the stomach for a longer period, so its stability in the stomach has also been studied. Phenolic compounds from apples showed stability in experiments in which they were incubated in an acidic medium similar to that of gastric juice [34] with a certain percentage of hydrolysis. After 4 h in the acidic medium, the percentage of hydrolysis of quercetin glycosides, the most abundant phenolic compounds in apple peel was only 4%, while the hydrolysis of procyanidins was somewhat higher, 27% [34]. In another study, after the incubation of apple peel polyphenols in simulated gastric juice without pepsin, the hydrolysis of flavonol glycosides was 3% and that of procyanidins was 27% [35]. It seems that low pH corresponds well to native forms of phenolic compounds even though a smaller percentage might hydrolyze due to low pH.

#### 4.1.2. Small Intestine

Passing from the stomach to the small intestine, phenolic compounds reach an environment of higher pH. Portions of them are still present in their native forms [5,23,24,26] (Figure 2). However, they can undergo some transformations or degradation due to the increased pH value. That is why we can also find the products of transformation/degradation of phenolic compounds as well as phase II metabolites in the small intestine (Figure 2). The following text reports the forms of the main phenolic subgroups from apples present in the small intestine.

*Flavan-3-ols:* In studies conducted on ileostomy patients after they consumed apple juice [26] or an apple smoothie [33], 90% [26] or 62% [33] of ingested oligomeric procyanidins were recovered in the ileal fluid, which means that high amounts of native oligomeric procyanidins reach the small intestine. However, their polymerization degree might decrease, and smaller units cleaved from the oligomeric compounds might be absorbed [26,33]. On the other hand, smaller molecule flavan-3-ols such as (+)-catechin and dimeric procyanidins (procyanidin B1 and B2) were not found in the ileal fluid [26], or (+)-catechin and (−)-epicatechin were found in low amounts [33], which suggests that they could have been absorbed [26] or degraded into unknown products [31]. Similarly, in vitro studies suggested that procyanidins and monomeric catechins from apples might degrade in the small intestine [5]. Furthermore, phase II metabolites (epi)catechin *O*-sulfates found after the consumption of a smoothie were the result of the absorption of monomers by enterocytes and metabolism, which resulted in the appearance of sulphated compounds [33]. According to the mentioned studies, native flavan-3-ols with higher molecular mass are present in the small intestine, however, with a lower degree of polymerization. Smaller molecules might degrade into unknown products, or they can be metabolized into phase II metabolites.

*Phenolic acids*: Studies of in vitro simulated digestion of apples showed that chlorogenic acid can be present in its native form in the small intestine, but a portion of it can isomerize. This has been shown by the identification of native chlorogenic acid and its isomers (neochlorogenic and cryptochlorogenic acid) after the intestinal phase of simulated digestion of apples [5]. Similarly, after human subjects ingested apple juice [26] or an apple smoothie [33], chlorogenic acids that originate from apples (4-caffeoylquinic acid (cryptochlorogenic acid) and 5-caffeoylquinic acid (chlorogenic acid)) were found in the ileal fluid, together with their isomers (1-caffeoylquinic acid and 3-caffeoylquinic acid (neochlorogenic acid)) [26,33]. The presence of 3-caffeoylquinic acid confirmed the isomerization of native chlorogenic acids from apples in the human small intestine [26] similar to in vitro studies. However, 1-caffeoylquinic acid could be the product of esterase activity of the enterocytes in the small intestine [26]. Native *p*-coumaroylquinic acids were also detected in the ileal fluid [26,33]. In addition, studies in humans revealed the presence of other metabolites of phenolic acids in the small intestine (d-(−)-quinic acid, methyl caffeate, and methyl *p*-coumarate) [26]. d-(−)-quinic acid can be the product of the degradation of 5- and 4-caffeoylquinic acids or *p*-coumaroylquinic acid from apples, a reaction that might be catalyzed by esterase activity [26,33]. Methyl caffeate and methyl *p*-coumarate might have been created in the liver and transferred to the small intestine via enterohepatic recirculation or they could be the results of the activity of the carboxyl esterase of enterocytes [26]. As the described studies have shown, phenolic acids from apples appear in the small intestine in the native form, in the form of biotransformed compounds such as isomers of chlorogenic acids, and in the form of metabolites.

*Dihydrochalcones:* Native dihydrochalcones (phloretin-2′-glucoside, phloretin-2′-xyloglucoside) are stable in the small intestine according to in vitro studies [5,31,36]. The previously mentioned study in humans supported the stability of dihydrochalcones since phloretin-2′-xyloglucoside was found in the ileal fluid after subjects consumed apple juice [26,33]. However, phloretin-2′-glucoside was not found in the ileal fluid, but its phase II metabolite phloretin-2′-glucuronide was [26,33]. It was suggested that phloretin-2′-glucoside in the digestive system can be cleaved into phloretin and glucose. An aglycon phloretin can then be absorbed. Its glucuronidation takes place in the liver after which phloretin-2′-glucuronide is created. A metabolite, phloretin-2′-glucuronide can reach the small intestine through the enterohepatic recirculation, or glucuronidation could take place in the enterocytes of the small intestine [26]. An aglycon phloretin has been found in the ileal fluid after the consumption of an apple smoothie [33]. As suggested, dihydrochalcones from apples can be present in the small intestine in their native form and in the form of phase II metabolites, and even aglycons can be present in a low amount.

*Anthocyanins:* In vitro studies suggest that anthocyanins from apples change form when passing from the stomach and low pH, in which they have the form of flavylium cation, into the small intestine and higher pH [4,5,23,24]. At the higher pH in the small intestine, they change into a colorless carbinol pseudo-base [37]. In addition to the influence of pH, the presence of initial low amounts of anthocyanins in the consumed food contributes to their loss in the small intestine. Since anthocyanins change form in the small intestine and their initial amounts in whole apples is usually low, they are not identified in their native form in the small intestine [23,24]. Similarly, in the study in humans who consumed apple juice, anthocyanins were not found in either the apple juice or the ileal fluid after the consumption of juice [26]. Accordingly, it can be suggested that due to their usually low amount in whole apples and the influence of a higher pH on the change in their native form, anthocyanins from apples will usually not be found in the small intestine in their native form.

*Flavonols*: Flavonols are found in the small intestine in their native form according to in vitro simulated digestion of apples [5,23,24,31]. However, they were not found in the ileal fluid after human subjects consumed apple juice [26], which might be the result of their low amount in the consumed juice. In contrast, after the human subjects consumed an apple smoothie, native flavonols were present in the ileal fluid [33] due to the higher amount of flavonols in the smoothie. It can be suggested that flavonols can be present in the native form in the small intestine. However, this amount depends on the amount in the consumed apple or apple product. Usually, apple peel contains higher amounts of flavonols. If the apple with the peel is consumed, that can lead to potentially higher detectable amounts of native flavonols in the small intestine, as it has been shown in earlier studies [5,6,23,24,25,31,32,33].

#### 4.1.3. Colon

The mixture of all forms of phenolic compounds in the small intestine mentioned earlier, such as native phenolic compounds, transformed compounds, and metabolites, reaches the colon and is present in the colon at least at the beginning phase (Figure 2). Furthermore, native phenolic compounds can be additionally released in the colon. This has been shown in an in vitro study [25]: insoluble material, which remained after in vitro oral, gastric, and duodenal phases of digestion, was subjected to the action of bacterial protease (suitable to hydrolyze insoluble material) and a cellulolytic enzyme mixture (to completely disrupt the whole-fruit matrix). This in vitro experiment simulated the phase of the digestion in the colon. In that experiment, amongst different native phenolic compounds released from whole apples or apple flesh, hydroxycinnamic acids were released in the highest amount. In the case of the colonic digestion of apple peel, flavonols were released in the highest amount [25]. However, during their passage through the colon, most of them were degraded into various catabolites due to fermentation by the present microbiota [27,38]. After 6 h of colonic fermentation of apple polyphenol extracts, a small percentage of native phenolic compounds were detected, only 0.4–0.5% of the initial amount. The amounts of catabolites were much higher [38]. Similarly, after the 24 h colonic fermentation of apple polyphenols, small amounts of native phenolic compounds from apples were found, but the amounts of catabolites were higher [27].

Various catabolites can be found after colonic fermentation. When three cultivars of commercial apples were fermented in vitro using a colonic fermentation experiment, catabolites belonging to phenylacetic acid derivatives, phenylpropionic acid derivatives, benzoic acid derivatives, and cinnamic acid derivatives [11] were found. The degradation of precursor native polyphenols started at 5 h of the fermentation and was completed after 24 h [11]. Similar catabolites belonging to the same groups of derivatives were reported after the colonic fermentation of the insoluble fraction remained after the in vitro gastrointestinal digestion of apple peel [32]. Examples of some catabolites are shown in Figure 2.

Chlorogenic acid, which is the most prevalent phenolic acid in apples, has relatively low absorption in the stomach and small intestine (33%) and passes to the colon at a high percentage [39,40]. Accordingly, its degradation in the colon might be important. Chlorogenic acid is hydrolyzed into caffeic and quinic acid by the activity of bacterial cinnamoyl esterase [39,41,42] and then to other catabolites. Its product caffeic acid has the potential for beneficial effects in the colon itself. The hydrolyzation with bacterial cinnamoyl esterase and the presence of caffeic acid in the colon gives additional positive value to chlorogenic acid from apples.

According to these studies, it seems that the most diverse mixture of phenolic compounds from apples could be present in the colon at the beginning of the passage through the colon (native compounds, metabolites, degradation products, and catabolites). Some of them can be absorbed. All these forms can have the potential for positive influence in the colon since they are present there. However, the time of passage through the colon eventually leads to biotransformation into catabolites. That is why special attention needs to be paid to catabolites. Since one of the major compounds in apples is chlorogenic acid, the presence of chlorogenic acid, its product caffeic acid, and the enzymes that hydrolyze chlorogenic acid, such as cinnamoyl esterase, might all be important and confer beneficial effects in the colon.

## 5. Beneficial Effects of Phenolic Compounds from Apples in the Digestive Tract

### 5.1. Beneficial Effects in the Stomach

**Figure 3:**
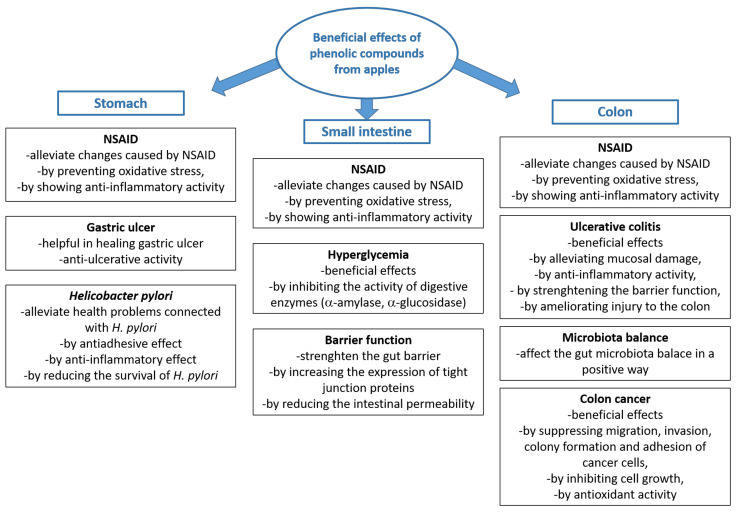
Beneficial effects of phenolic compounds from apples in the digestive tract [2,3,11,17,21,27,34,35,38,40,43,44,45,46,47,48,49,50,51,52,53,54,55,56,57,58,59,60,61,62,63,64,65,66,67] (NSAID—nonsteroidal anti-inflammatory drug).

It can be suggested that native phenolic compounds from apples are the forms that have the potential for bioactive effects in the stomach. Some of the effects in the stomach are shown in Figure 3.

Gastric mucosa can be damaged by the chronic administration of nonsteroidal anti-inflammatory drugs (NSAIDs). It has been shown that indomethacin, an NSAID, increases the oxidative stress and lipid peroxidation in gastric and intestinal cells [34]. Some potential beneficial effects of phenolic compounds from apples in the stomach are to alleviate changes caused by NSAIDs, which was shown in [34]. In that study, rats received 175 to 350 mg/kg of apple peel extract and then indomethacin (40 mg/kg). Apple peel polyphenols protected gastric mucosa of rats after gastrointestinal damage induced by indomethacin by decreasing the damage to the gastric mucosa [34]. The protection was shown through their protection of gastric mucosa against oxidative stress (preventing the concentration of malondialdehyde to increase) and through their anti-inflammatory activity (preventing neutrophil infiltration into the mucosa) [34].

Phenolic compounds from apples can be helpful in the healing of gastric ulcers. In studies in animals, where gastric ulcers were induced by acidified ethanol (150 mM HCl/ethanol, 40:60 *v*/*v*) [43,44], apple juice (3 mL), phenolic compounds from apples (5 to 20 mg), apple pectin [43], or phenolic compounds from apples (20 mg) [40] given to animals before the acidified ethanol [43,44] showed not only anti-ulcerative activity [43] but also a pro-ulcerative effect [43,44]. More precisely, the suppression of ulcer induction was shown for the apple juice, for lower amounts of 5 to 10 mg of semi-purified phenolics from apples, and for pectin [43], but a higher amount of 20 mg of semi-purified phenolics showed a pro-ulcerative effect [43,44]. Even though the higher amount of semi-purified phenolics from apples showed a pro-ulcerative effect, the authors suggested that the natural concentrations of phenolics in the fruit or juice seem to be appropriate for beneficial effects [43]. However, care should be taken with purified extracts [43]. The main factors in anti-ulcerative activity were phenolic compounds from apples, while pectin contributed an additional effect [43]. The main mechanism behind the anti-ulcer activity might be the radical scavenging activity of phenolics [43]. In another study conducted in rats, where gastric ulcers were induced with indomethacin (50 mg/kg), chlorogenic acid (50 to 100 mg/kg) was given to animals for 14 days before indomethacin administration [45]. That study also showed the protective effects of chlorogenic acid against mucosa damage [45].

Phenolic compounds from apples can help alleviate health problems connected with *Helicobacter pylori (H. pylori).* This bacterium can cause some gastrointestinal disorders that can lead to gastric ulcers or gastric carcinoma. After penetration into the mucin layer, *H. pylori* adheres to epithelial gastric cells, where it can cause oxidative stress, inflammation, and cell death [35]. An extract of apple peel showed in an in vitro study in cells (HeLa) that it can have an antiadhesive effect against *H. pylori*. It reduced *H. pylori* attachment by 20–60% [35]. In another in vitro study, apple peel polyphenols inhibited multiplication of two *H. pylori* strains [46]. In a study in mice, a bacterial suspension with *H. pylori* was given to the mice four times in eight days. Apple peel polyphenols (150 or 300 mg/kg/day) were given to mice for three additional weeks. Apple peel polyphenols showed their inhibitory effect on *H. pylori* attachment and an anti-inflammatory effect [35]. Moreover, *H. pylori* can produce urease, which can generate ammonia. Ammonia neutralizes gastric acidity and creates a neutral environment around the bacterium [47]. High-molecular-weight polyphenols from apple peel are recognized as active compounds. They have been shown to inhibit the activity of urease [47], which can reduce the survival of *H pylori* in the stomach.

Nitric oxide (^·^NO) is a molecule that can have an important biological function in the stomach, such as via antimicrobial activity or the increase in mucus production. It can be created from nitrates that we consume with food, especially leafy vegetables. Nitrates from food can be reduced to nitrites by the bacteria in the mouth. Further acidification of nitrites in the stomach generates ^·^NO. Studies have shown that apple peel has the potential to promote ^·^NO bioavailability in the stomach [48].

Apple polyphenols can be helpful in the stomach by decreasing the risk of the side effects caused by consuming NSAIDs. Moreover, it has been suggested that phenolic compounds from apples should be evaluated as nutraceuticals to decrease the risk of NSAID consumption [34]. Another way of using apple phenolics in the stomach is against health problems related to gastric ulcers or *H. pylori* infections. Since mostly native forms of phenolic compounds from apples are present in the stomach, those forms have the potential for the mentioned positive effects. However, most of the described studies ascribe effects to the apple phenolic compounds or apples in general. Further studies might reveal compounds or a group of phenolic compounds with the bioactivity in the stomach.

### 5.2. Beneficial Effects in the Small Intestine

The mentioned in vitro studies of simulated digestion and the studies in humans showed that phenolic compounds from apples can be found in the small intestine in the native form as they appear in fruit, in the form of biotransformed compounds and in the form of phase II metabolites. These forms can be active compounds with potential bioactivities in the small intestine. Here, we mention some of the conditions in the small intestine that can benefit from consuming apples (Figure 3).

Hyperglycemia is a condition of an increased level of glucose in the blood and is associated with diabetes. Digestive enzymes such as α-amylase in the mouth and small intestine or α-glucosidase in the brush border of the small intestine are important for starch digestion, the product of which is glucose. Glucose is then absorbed from the small intestine into the blood. Compounds that can inhibit digestive enzyme activities, can inhibit the digestion of starch and the production of glucose, which in turn can be helpful in hyperglycemia due to the potential to lower or maintain the level of glucose in the blood. There are some anti-diabetic drugs such as acarbose that have this ability, to interfere with carbohydrate digestive enzymes. However, these drugs might have side effects, which is why studies are trying to find natural compounds with a similar role, to inhibit digestive enzymes. In the following studies, phenolic compounds from apples have shown the potential to inhibit the activity of digestive enzymes (α-amylase, α-glucosidase). It was shown that young apple polyphenols inhibit porcine pancreatic α-amylase activity in in vitro studies [17,21,49,50]. The inhibition depended on the apple variety [17,21]. Phenolic compounds from apples that correlated positively with the inhibition were *p*-coumaroylquinic acid, 5-caffeoylquinic acid (chlorogenic acid), procyanidin B1, and quercetin-3-glucoside [21]. In studies where the inhibition of α-amylase was conducted with young apple polyphenol extract and individual polyphenols that can usually be found in apples, it was also shown that young apple polyphenols inhibited enzyme activity, and the strength of this inhibition follows the order chlorogenic acid > caffeic acid > epicatechin [49]. Furthermore, it was suggested that the inhibition was caused by the binding of the polyphenols to the enzymes according to the order chlorogenic acid > caffeic acid > epicatechin [50]. The bonds that can be created are H bonds and hydrophobic interactions [50]. Furthermore, phenolic compounds from young apples inhibit the activity of α-glucosidase [17,51], which is also dependent on the apple variety [17]. The inhibition of enzyme activity is caused by the binding interactions [51]. Besides in vitro studies, studies have been conducted in animals, which suggest that apple polyphenols decrease blood glucose levels. In a study in mice, the acute hypoglycemic effect of young apple polyphenols and individual polyphenols was studied [17]. Mice were fed with a combination of starch (5 g/kg bw) and young apple phenolic compounds (150 mg/kg bw), phloretin-2′-glucoside (150 mg/kg bw), and chlorogenic acid (150 mg/kg bw). Phenolic compounds from young apples decreased the peak blood glucose level by 13% [17]. In addition, a one-week intervention hypoglycemic effect was also studied. In that study, mice were given young apple phenolic compounds (150 mg/kg bw), phloretin-2′-glucoside (150 mg/kg bw), and chlorogenic acid (150 mg/kg bw) for 6 days. Then, mice were treated with starch (5 g/kg bw). Phenolic compounds from young apples decreased the peak of blood glucose by 7% [17]. In addition, in a randomized double-blind placebo-controlled trial, after a chronic administration of apple polyphenols to human subjects for 12 weeks, it was shown that impaired glucose tolerance was significantly improved in high-normal and borderline subjects [52]. The mentioned studies highlighted some of the native compounds from apples with an ability to inhibit the activity of digestive enzymes and to be active compounds with bioactive effects. But studies have also mentioned the collective effects of all phenolic compounds from apples.

As already mentioned, chronic administration of nonsteroidal anti-inflammatory drugs (NSAIDs) can be associated with adverse effects in the gastrointestinal tract [34]. They can increase lipid peroxidation and the production of reactive oxygen species in the gastrointestinal mucosa [34], increase the activity of pro-oxidant enzymes, and decrease the activity of antioxidant enzymes [34]. This can affect intestinal mucosa. In vitro studies [53] and studies in animals [34] have shown the potential beneficial effects of apple polyphenols in the intestinal mucosa after treatment with NSAIDs. In a study on rats, the rats received 175 to 350 mg/kg of apple peel extract, and then indomethacin (40 mg/kg), an NSAID. The use of indomethacin caused damage to the duodenal mucosa, even though much less damage than that in the gastric mucosa. Apple peel polyphenols attenuated those changes [34] by protecting the intestinal mucosa from oxidative stress (by decreasing the activity of the pro-oxidant enzyme myeloperoxidase). They also prevented lipid peroxidation (visible by a decreased malondialdehyde concentration) and showed their anti-inflammatory activity [34]. The described studies ascribed the effects to phenolic compounds present in apples.

A gut barrier is formed from a layer of epithelial cells positioned between the intestinal lumen and the mucosal tissue, joined together by a tight junction. The tight junction is composed of transmembrane proteins and some other molecules. That layer has a role in regulating the flow and permeation of molecules. If this barrier function is damaged, the permeability can increase, which allows harmful pathogenic organisms, harmful molecules, or microbial fragments to enter the bloodstream [54]. The condition of increased permeability of the gut barrier is called “a leaky gut”. Apple polyphenols have the potential to strengthen the gut barrier by increasing the expression of tight junction proteins [2,3] and by reducing the intestinal permeability [3,34]. In a study in rats, gastrointestinal damage was induced by indomethacin (40 mg/kg), and rats were pretreated with apple peel polyphenols (175–350 mg/kg) [34]. It was shown that in animals that received indomethacin, the permeability to sucrose increased three times in comparison to the control, and the permeability to lactulose/mannitol increased twice in comparison to the control. Apple peel polyphenols prevented the increase in gastrointestinal permeability [34]. In a study in mice [2], mice were fed with a high-fat diet together with phloretin-2′-glucoside (10, 30 and 60 mg/kg bw) daily for 12 weeks. The supplementation of the diet with phloretin-2′-glucoside showed a positive effect on the mRNA expression of the tight junction and mucus genes and enhanced barrier functionality [2]. In a study in which mice received dextran sulfate sodium, apple peel extract (500 mg/kg bw, daily) was also given to mice for 7 days [3]. Apple polyphenols strengthened the intestinal barrier by increasing the expression of tight junction proteins and reducing the intestinal permeability. Mice received patulin (5 mg/kg/day), a mycotoxin that can damage the gut barrier, together with lignin-based cross-linked particles loaded with chlorogenic acid (2 g/kg bw) for three weeks [55]. The patulin disrupted the intestinal barrier function, while the particles with chlorogenic acid reduced the damage to the barrier function [55]. The beneficial effects in those studies were ascribed to apple polyphenols in general and to some individual native phenolics.

Phenolic compounds from apples can potentially be beneficial in overcoming hyperglycemia, in alleviating side effects after chronic administration of nonsteroidal anti-inflammatory drugs, and in preserving the barrier function in the small intestine, which can be connected to many diseases. Usually, those effects are ascribed to particular native phenolic compounds from apples or to native apple polyphenols in general. In studies conducted on animals, other forms are also present in the small intestine, such as biotransformed compounds or metabolites that also have a potential for bioactive effects. Since there are many forms of phenolic compounds present in the small intestine, further studies should focus on determining real active compounds with potential for beneficial effects.

### 5.3. Beneficial Effects in the Colon

The most diverse forms of phenolic compounds can be present in the colon, but their presence depends on the amount of time of digestion in the colon. All forms of phenolic compounds that are present in the small intestine (native compounds, bio-transformed compounds, or phase II metabolites) can pass to the colon and be present in the colon in the beginning phase of digestion. However, with time, catabolites become the dominant compounds. All the mentioned forms of phenolic compounds can potentially be active forms with bioactive effects. Some of the potentially positive effects of phenolic compounds from apples in the colon are presented in Figure 3.

Chronic administration of nonsteroidal anti-inflammatory drugs (NSAIDs) can affect colon mucosa as well [34,53]. In rats, the use of indomethacin (40 mg/kg) damaged the colon mucosa. However, apple peel extract (175 to 350 mg/kg) had beneficial effects by showing antioxidant activity, by preventing lipid peroxidation, and by exhibiting anti-inflammatory activity [34]. In an in vitro study in Caco-2-cells, indomethacin caused several effects, including cell loss, the intracellular production of reactive oxygen species (superoxide), the elevation of pro-oxidant enzyme (xanthine oxidase), the increase in lipid peroxidation, and the decline in cell viability [53]. Apple peel polyphenols prevent these effects [53].

Ulcerative colitis (an inflammatory bowel disease) is a chronic disease that can affect the large intestine. It can cause inflammation along with abdominal pain, bloody stool, and weight loss [3,56]. Patients with ulcerative colitis may have damaged the barrier function of the intestine, which increases permeability and allows harmful pathogenic organisms to enter the blood. Studies in animals have shown the potential beneficial effects of apple polyphenols on ulcerative colitis [3,56]. In a study where ulcerative colitis was induced in mice by dextran sulfate sodium, mice also received an apple peel extract (500 mg/kg bw, daily) for 7 days [3]. The apple polyphenols alleviated mucosal damage in the colon, inhibited the inflammation in the colon, regulated the gut microbiota, and strengthened the intestinal barrier. All this shows a potential positive influence of apple polyphenols on colitis [3]. In another study, ulcerative colitis was induced in rats by trinitrobenzensulphonic acid (20 mg) [56]. Rats were treated with apple polyphenols (10^−4^ M) for 14 days by rectal administration of apple polyphenols. Apple extract reduced the severity of colitis, ameliorated macroscopic injury to the colon, partially restored the colon thickening, and restored all biomarkers at the baseline level [56]. The mentioned studies ascribed the beneficial effects to the phenolic compounds present in apples.

The majority of microorganisms that make up the human microbiota are found in the colon (10^12^ cells per gram of intestinal content) with four dominant bacterial phyla Bacillota (Firmicutes), Bacteroidota (Bacteroidetes), Actinomycetota (Actinobacteria), and Pseudomonadota (Proteobacteria). An imbalance in the human microbiota can affect many diseases, and so it is intensively studied. Studies are trying to find out how our diet affects the human microbiota and what kinds of consequences this can have on human health. Phenolic compounds can affect the balance of microorganisms in the colon in a positive way. The influence of phenolic compounds on the gut microbiota has been described in many papers, and an example is a review by Dueñas et al. [57]. Phenolic compounds from apples can also affect the gut microbiota balance in a positive way. It was mentioned that apple polyphenols can increase the abundance of anaerobic and facultatively anaerobic bacteria, which is positive due to anaerobic conditions in the colon, and deplete aerobic bacteria [58]. Several studies have associated apple polyphenols with the increased abundance of Verrucomicrobia, specifically with the increase in the relative abundance of *Akkermansia* [58,59,60]. *Akkermansia* can be connected to the inhibition of over-growth of potentially pathogenic species [58], and it is a beneficial microbe [59]. Furthermore, a higher ratio of Firmicutes/Bacteroidetes is associated with obesity, and apple polyphenols can decrease this ratio [2,60]. Specifically, in a study in which mice were fed a high-fat diet and treated with 125 or 500 mg/kg bw per day of apple polyphenol extract for 12 weeks [59] and in a study where mice were fed a high-carbohydrate diet, high-fructose, and high-sucrose diet and were also fed 125 or 500 mg/kg bw of apple polyphenol extract [58], the apple polyphenol extract regulated the diversity of the gut microbiota composition and increased the relative abundance of *Akkermansia* [58,59]. In addition, the relative abundance of Firmicutes was decreased but not that of Bacteroidetes [58], which decreases the ratio of Firmicutes/Bacteroidetes. In another study, after mice were fed a high-fat diet supplemented with apple peel, apple extract, or whole apples for four weeks, the microbiota changed [60], whole apples increased the levels of *Akkermansia*, and the ratio of Firmicutes/Bacteroidetes was lowered [60]. The supplementation of a high-fat diet for mice with phloretin-2′-glucoside rebuilt the gut microbiota homeostasis, increased the levels of produced short-chain fatty acids in the cecum [2], and normalized the ratio of Firmicutes/Bacteroidetes. Since Firmicutes and Bacteroidetes are important gut microbes, their increase or decrease caused by different factors represents a disturbance in the gut microbiota [3]. In a study where mice received dextran sulfate sodium but also apple peel extract (500 mg/kg bw, daily) for 7 days [3], the dextran sulfate sodium disturbed the Firmicutes and Bacteroidetes by reducing the relative abundance of Firmicutes and promoting the relative abundance of Bacteroidetes [3]. This lowered the ratio of Firmicutes/Bacteroidetes [3]. Apple polyphenols reversed that effect and improved the ratio by reducing the abundance of Bacteroidetes [3]. Apple polyphenols also reduced the abundance of *Escherichia-Shigella*, *Bacteroides,* and *Parasutterella* [3]. Some studies have connected apple polyphenols to an increase in *Lactobacillus* and *Bifidobacterium* [61,62], which can inhibit the growth of some pathogens [11]. When rats were fed extraction juices from apples for four weeks, the fecal count of *Lactobacillus* and *Bifidobacterium* increased [61]. In a study in pigs, the pigs were fed with a basal diet supplemented with 400 and 800 mg/kg of apple polyphenols for 49 days [62]. The apple polyphenols increased *Lactobacillus*, *Bifidobacterium,* and *Bacillus*, which might improve the intestinal immune barrier [62]. After the colonic fermentation of three apple varieties, the presence of fermented apples significantly changed the bacterial diversity [11]. In particular, it increased the *Bifidobacterium* relative abundance [11]. Opposite to that, in a study by Li et al. [59], the levels of *Lactobacillus* decreased, which might affect bile acid metabolism, but these effects need further investigation [59]. The increase in Proteobacteria, which includes some pathogenic bacteria, was also shown [11,58]. In a study by Koutsos et al. [11], where the colonic fermentation of apples was studied, the Proteobacteria abundance increased. However, fortunately, the *Enterobacteriaceae* family, which includes many pathogenic bacteria (*Escherichia*, *Salmonella*, and *Yersinia*) and is a major member of Proteobacteria, did not show an increase [11]. As we have surveyed, many mentioned studies connected apple polyphenols with beneficial influence on the diversity and balance of the gut microbiota, but more studies are needed to confirm these effects.

Colon cancer is one of the most common diseases in the world. The prevention of this disease is important and could lead to a potential lower rate of mortality [63]. Researchers are trying to find the evidence for natural compounds that can be helpful in lowering the risk of developing colon cancer. Phenolic compounds might have that capability. Phenolic compounds from apples have been studied for beneficial effects in colon cancer [38,64,65,66]. Studies conducted on colon cancer cell lines in vitro have shown that apple phenols have the potential for beneficial effects in the colon [38,65,66]. In a study by Hung et al. [66], apple polyphenols suppressed migration, invasion, colony formation, and adhesion of human colon cancer cells in a concentration-dependent manner. Moreover, annurca apple polyphenols were incubated with colorectal cancer cells, and they decreased the cell viability and induced apoptosis in a concentration- and time-dependent manner [65]. In a study by Bellion et al. [38], colon cancer cells were incubated with apple juice extract and with supernatant obtained after the colonic fermentation of apple juice extract. The fermented apple juice extract exhibited antioxidant activity in colon cells but less than that for nonfermented apple extract. Antioxidant activity was shown through the decreased levels of reactive oxygen species and decreased oxidative DNA damage [38]. According to this established antioxidant activity, the researchers suggested that phenolic compounds and metabolites can protect the colon from oxidative stress and ROS-mediated diseases since phenolic compounds and metabolites are present in the fermented sample [38]. In a similar study, colon adenoma and carcinoma-derived cells were treated with apple extracts and with extract obtained after the fermentation of apple polyphenols with human fecal flora for 24 h. The cells were incubated with those extracts for 24, 48, and 72 h [27]. All the extracts inhibited cell growth, but the apple extracts more so than the fermented apple extract. It was suggested that quercetin derivatives in apples were more potent inhibitors of cell growth, and in the fermented sample, catabolites were recognized as active compounds such as 3,4-dihydroxyphenylpropionic acid and phloroglucin [27]. In these in vitro studies, the effects were ascribed to phenolic compounds from apples in general, to a specific group of native phenolic compounds, and also to apple juice extract after colonic fermentation that contains not just native phenolic compounds but also catabolites. This highlights the role of not only native phenolic compounds but also all other forms that are present in the colon. The effectiveness of phenolic compounds from apples has also been studied in animals. Mice were treated with human colon cancer cells, which were treated or not with apple polyphenols [66]. After 6 weeks, implanted colon cancer cells strongly attached to the colon tissue and formed numerous tumors in mice. Cells that were treated with apple polyphenols rarely attached to the colon tissue and formed fewer numbers of tumors [66]. In a study by Bars-Cortina et al. [64], rats were treated with the carcinogen azoxymethane (15 mg/kg) once per week in a two-week period. Rats received a standard diet or a standard diet enriched with apple polyphenols (3553 mg of lyophilized apple flesh/kg rat per day) or apple cyanidin-3-galactoside for 8 or 14 weeks. The results suggested the effects of apple polyphenols and cyanidin-3-galactoside against colon carcinogenesis, retarding the appearance of precancerous markers [64]. It was concluded that apple phenols showed potential chemopreventive effects at a daily dose equivalent to the consumption of one apple (without peel) per day in humans [64]. In a study by Barth et al. [67], rats were treated with 1,2-dimethylhydrazine to induce colon carcinogenesis. Rats received cloudy apple juice, a polyphenol fraction from apple juice, or a cloudy fraction for seven weeks, starting one week before treatment with 1,2-dimethylhydrazine (20 mg/kg bw four times at one-week intervals). The genotoxicity of colonocytes was reduced by cloudy apple juice. The cell proliferation decreased by cloudy apple juice, polyphenol fraction, and a combination of a polyphenol fraction and a cloudy fraction [67]. In this study, besides the polyphenol fraction, the cloudy fraction was also identified as a bioactive fraction [67]. According to these results, the mentioned studies in animals highlighted not only the role of phenolic compounds from apples in general but also the matrix of the food such as a cloudy fraction of apple juice. On the other hand, Lhoste et al. [68] used a model of human microbiota-associated rats, germ-free rats, or conventional rats. Rats were fed a human-type diet and injected with 1-2 dimethylhydrazine (50 mg/kg). The number and size of aberrant crypt foci, which are the earliest identifiable lesions in colon carcinogenesis, were higher than in the conventional rats and germ-free rats. The aberrant crypt foci number and multiplicity were not reduced by feeding rats with apple proanthocyanidin extract (0.001 and 0.01% in drinking water), and they were higher with 0.1% of the extract in drinking water. According to this, it might be concluded that apple proanthocyanidins do not have any protective effects. However, the authors mentioned the possible antagonistic effect of the gut microbiota on the potentially protective effects of apple proanthocyanidins. Furthermore, they suggested the importance of a proper dose of proanthocyanidins that should be given to animals in the experiment. The proper dose should be determined according to the intake in humans. The average daily intake of proanthocyanidins in humans is evaluated to be 1 mg/day/kg bw, and they suggested that even an intake of 10 mg/day/kg bw will probably not be exceeded in extreme situations. In this study in rats, 0.01% procyanidins reached 10 mg/day/kg bw, and these higher doses of proanthocyanidins might be the reason for the lack of protective effects [68].

Phenolic compounds from apples can be helpful in the colon in alleviating side effects after chronic administration of nonsteroidal anti-inflammatory drugs or in keeping the microbiota balance. Phenolic compounds from apples might be a new strategy for the treatment of ulcerative colitis, and beneficial effects have been shown in colon cancer as well. Most of the studies ascribed the bioactive effects to phenolic compounds from apples in general, while some mentioned specific native phenolic compounds or specific metabolites or catabolites. In studies examining the effects of phenolic compounds from apples in the colon using animals, or in studies that used samples after the colonic fermentation, catabolites are present, which might highlight the role of catabolites as active compounds as well. More studies are necessary to discover which are the real active compounds with bioactive effects in the colon, focusing on catabolites since their concentration increases with the time of fermentation. The concentrations that show potential beneficial effects should also be investigated as well as the role of the food matrix.

## 6. The Influence of the Food Matrix

Dietary fibers are an essential part of apples. In a total amount of dietary fibers of 2.4–2.6 g/100 g, soluble fibers are represented in 0.9–1.6 g/100 g, and insoluble fibers in 1-1.5 g/100 g [11]. Those food components can show bioactivities as well. Apple pomace is a byproduct obtained after processing apple juice. In a study by Kosmala et al. [69], apple pomace contained 61% dietary fiber and 0.23% phenolic compounds. Phenolic compounds were removed with ethanol or ethanol/acetone. Pomace after extraction contained 66% dietary fibers with 0.1% polyphenols (after the extraction with ethanol) or 67% dietary fibers with 0.01% polyphenols (after the ethanol/acetone extraction). These preparations were included in an air-dried feed in the amount of 5%, which was used to feed rats for four weeks [69]. Pomace rich in fiber had beneficial effects in the digestive tract of these rats, which was shown in the increased ileal digesta hydration and in the increased cecal short-chain fatty acid concentration. Polyphenols, mainly flavonoids, showed a minor effect [69]. One of the soluble dietary fibers, pectin, was also recognized as an important constituent of apples with potential bioactive effects. In a study by Licht et al. [70], rats were fed with whole apples (10 g/day), apple puree (10 g/day), cloudy apple juice (8 mL/day), clear apple juice (8 mL/day), pomace (0.5 g/day), or 0.33 or 3.3% apple pectin/day for fourteen weeks. In addition, rats were fed with whole apples (10 g/day) or 7% apple pectin/day for four weeks. It was suggested that apples have health-promoting effects on the intestinal microbiota; however, this effect can mainly be explained by the presence of pectin [70]. The experiment also showed an increase in *Clostridiales* abundance and butyrate concentration, which is considered beneficial for gut health [64]. Moreover, it has been shown in rats, that pectin, if taken together with high polyphenol freeze-dried apples, has more bioactive effectiveness than apple itself [71]. Apple polysaccharides (with a monosaccharide composition consisting of rhamnose, galacturonic acid, glucose, and galactose, in molar ratios of 1.00:20.30:8.50:8.03, respectively) prevented mice from exhibiting colitis-associated colorectal cancer by regulating intestinal flora disorder [72]. Since dietary fibers, pectin, or polysaccharides show potential beneficial effects in the digestive tract, the influence of the apple matrix should be studied further to see if it affects the bioactivities of phenolic compounds from apples.

## 7. Conclusions

Apples, as a fruit consumed in a daily diet and therefore a good source of phenolic compounds, have a real potential to be helpful for human health. The digestive tract represents the first contact of the human organism with these phenolic compounds. Phenolic compounds from apples are present in all parts of the digestive tract in relatively high amounts. This gives them an opportunity to confer beneficial effects directly in the digestive tract. There are many studies showing conditions that can benefit from consuming apples. In the stomach, those are gastric ulcer or infections caused by *Helicobacter pylori*. In the small intestine, studies are mentioning beneficial effects of apple polyphenols for strengthening the barrier function or in hyperglycemia. In the colon, apple polyphenols have the potential to show beneficial effects against colon cancer, ulcerative colitis, or in establishing the microbiota balance in a positive way. In the stomach, small intestine, and colon, apple polyphenols can be helpful in mitigating the consequences of chronic administration of nonsteroidal anti-inflammatory drugs. The most likely compounds that can show beneficial effects in the stomach are native polyphenols. For the small intestine, those are native compounds, products of the biotransformation of phenolic compounds, or phase II metabolites. All of these forms can pass to the colon; however, in time, they are metabolized into various catabolites. All of these forms have the potential for beneficial effects in the colon. But it is still not clear which are active compounds from apples, what influence the matrix of apples may have, or whether there are common interwoven actions of phenolic compounds and the matrix. All this should be considered in future studies.

## 8. Future Directions

Here are some suggestions for investigating the beneficial effects of phenolic compounds from apples in the digestive tract.

Distinguishing between different forms of phenolic compounds that are actually present in certain parts of the digestive tract and their specific activity should be considered more when explaining the effects of polyphenols from apples. It seems that native forms of phenolic compounds have a potential to show beneficial effects in the stomach and small intestine. Catabolites might be more important in the colon, even though the role of native compounds should not be ignored. The role of phase II metabolites in the small intestine could also be examined.

Apart from forms of phenolic compounds in the digestive tract, the time of digestion can affect bioactivities. Namely, the release of phenolic compounds from the food matrix, or their biotransformation, depends on the time of digestion. The influence of time on the release or the degradation of phenolic compounds in different phases of digestion require additional studies.

The food matrix is recognized as important in explaining the bioactive effects of phenolic compounds. In particular, the significance of the apple matrix requires additional studies. Finally, studies in humans should explain the real-world effects of phenolic compounds from apples in the digestive tract.

## Data Availability

The data presented in this study are available in Supplementary Materials Table S1.

## Supplementary Materials

The following supporting information can be downloaded at: https://www.mdpi.com/article/10.3390/molecules29030568/s1, Table S1: The amounts of native phenolic compounds from apples released in different phases of the digestion (in vitro simulated digestion) and in the colon from insoluble matrix after in vitro digestion.
