# Supplementary material for: Phenolic Compounds from Apples: From Natural Fruits to the Beneficial Effects in the Digestive System
Source: Molecules. 2024 Jan 23;29(3):568. doi: 10.3390/molecules29030568 (PMC10856038; doi:10.3390/molecules29030568)
Supplement: Supplementary file 1 [file molecules-29-00568-s001.zip › molecules-2791234-supplementary.pdf]

Supplementary Materials

Phenolic Compounds from Apples: from Natural Fruits to the Beneficial Effects in the Digestive System

Lidija Jakobek [1,*], and Petra Matić [1]

Table S1. The amounts of native phenolic compounds from apples released in different phases of the digestion (*in vitro* simulated digestion) and in the colon from insoluble matrix after *in vitro* digestion.

Phenolic	Apple	Before	Salivary	Gastric	Intestinal	Colon*	Reference
**Flavonols**	Peel (mg/100 g fw)	72-193	6-76	7-68	14-77		23
	Peel (mg/100 g fw)	22		11	13		24
	Peel (rutin) (mg/100 g fw)	10-44	4-15	7-29	5-21		6
	Flesh (mg/100 g fw)	2		0.8	2		24
	Flesh (rutin) (mg/100 g fw)	1-11	0.4-4	0.7-7	0.5-5		6
	Whole apple (mg/100 g fw)	11-15		10-15	12-19		5
	Flesh (μg/g dw)*				0.1-1.9	0.3-3	25
	Peel (μg/g dw) *				52-93	47-78	25
	Whole apple (μg/g dw) *				8-59	2-20	25
**Phenolic acids**	Peel (mg/100 g fw)	36-58	3-23	8-23	0-6		23
	Peel (mg/100 g fw)	14		4	3		24
	Flesh (mg/100 g fw)	30-41	5-12	12-21	1-4		23
	Flesh (mg/100 g fw)	3		1	1		24
	Whole apple (mg/100 g fw)	13-18		11-18	5-9		5
	Flesh (μg/g dw) *				2.1-46	3-120	25
	Peel (μg/g dw) *				1-14	5-62	25
	Whole apple (μg/g dw) *				83-120	46-113	25
**Dihydrochalcon**	Peel (mg/100 g fw)	1-8	0-3	0-2	0-4		23
	Peel (mg/100 g fw)	4		1			24
	Peel (phloridzin) (mg/100 g fw)	5-68	1-18	2-27	2-31		6
	Flesh (mg/100 g fw)	0.3-1	0.1	0.3-0.5	0-0.3		23
	Flesh (mg/100 g fw)	1		0.6			24
	Flesh (phloridzin) (mg/100 g fw)	1-10	0.3-3	0.4-4	0.5-5		6
	Whole apple (mg/100 g fw)	2-3		2	3-5		5
	Flesh (μg/g dw) *				0.1-19	0.3-11	25
	Peel (μg/g dw) *				4-19	3-29	25
	Whole apple (μg/g dw) *				9-38	3-18	25
**Flavan-3-ols**	Peel (mg/100 g fw)	40-156	2-64	7-85	0-4		23
	Peel (mg/100 g fw)	20		6			24
	Peel (procyanidins) (mg/100 g fw)	6-44	2-15	2-17	3-25		6
	Flesh (mg/100 g fw)	11-21	0.5-1.2	3-4			23
	Flesh (mg/100 g fw)	3		4			24
	Flesh (procyanidins) (mg/100 g fw)	16-79	6-28	6-32	9-44		6
	Whole apple (mg/100 g fw)	10-15		6-12			5
	Flesh (μg/g dw) *				0-0.003	0.1-6	25
	Peel (μg/g dw) *				0	2-16	25
	Whole apple (μg/g dw) *				0.04-0.6	1-10	25
**Anthocyanins**	Peel (mg/100 g fw)	0.2-20	0-4	0-6			23
	Peel (mg/100 g fw)	2		4			24

**Total phenolic compounds**	Peel (mg/100 g fw)	153-424	11-167	22-182	15-91		23
	Peel (mg/100 g fw)	62		22	16		24
	Peel (µg/g dw)				75-100		25
	Flesh (mg/100 g fw)	48-63	6-13	17-25	1-4		23
	Flesh (mg/100 g fw)	9		6	3		24
	Flesh (µg/g dw)				2.3-66		25
	Whole apple (mg/100 g fw)	36-52		29-47	18-27		5
	Whole apple (µg/g dw)				104-196		25
	Flesh (µg/g dw) *					4-138	25
	Peel (µg/g dw) *					68-192	25
	Whole apple (µg/g dw) *					53-166	25

*The release of phenolic compounds in the colon from indigestible insoluble material remained after the in vitro gastrointestinal digestion. Experiments were conducted with the help of pronase to simulate the action of bacterial protease, and with the help of Viscozyme L (cellulolytic enzyme mixture) to completely disrupt the apple matrix [27]
